# Insight into the mechanical properties and the sustainable application of recycled permeable concrete: Green low-carbon concrete technology

**DOI:** 10.1371/journal.pone.0318684

**Published:** 2025-02-06

**Authors:** Shaojian Xia, Lei Peng, Wu Zhang, Hongliang Zhu, Jing Li

**Affiliations:** 1 China Railway Seventh Group Co., Ltd., Zhengzhou, China; 2 School of Architectural Engineering, Guangzhou Vocational University of Science and Technology, Guangzhou, China; Shandong University of Technology, CHINA

## Abstract

The performance optimization of reclaimed permeable concrete helps to reduce the energy consumption of building materials preparation and promotes green and sustainable development. To further adjust the ratio of reclaimed permeable concrete and optimize the mechanical properties of reclaimed permeable concrete, the influence of aggregate particle size distribution on the mechanical properties of reclaimed permeable concrete is analyzed. In the analysis of the effect of particle size distribution on the mechanical properties of recycled aggregate permeable concrete, the design experiment was examined. The experiment showed that when the water-cement ratio was 0.28/0.30/0.32 and the porosity was 10%, the increase in permeability coefficient was 28.1%/50.9%/29.0%. When the recycled permeable concrete had a water-cement ratio of 0.30 and a particle grading of 8:2 at a target porosity of 10%, the average permeability coefficient was the lowest, only 1.23mm/s. The circulating permeable concrete of the study design met the requirement of the permeability coefficient under the CJJ/T1352009 standard, which was not less than 1 mm/s. The extant research on the influence of aggregate grading and other parameters on the performance of recycled permeable concrete indicates that the ratio of recycled aggregate permeable concrete can be optimized. Furthermore, the customization of concrete ratio according to the needs of different buildings can effectively reduce the manufacturing cost of building materials.

## 1. Introduction

The continuous development of the social economy has led to a notable expansion in the use of building materials, particularly concrete, as cities undergo further urban expansion [1]. The sand and stone reserves surrounding the city have been significantly depleted. However, the demand for natural aggregates in the construction industry exhibits an annual increase rather than a decrease, resulting in a substantial imbalance in the supply and demand relationship [2]. In the process of deepening urbanization, various types of buildings are constantly being demolished and rebuilt, resulting in a large amount of construction waste. These construction wastes are difficult to handle, and most of them are directly transported to the outskirts for stacking in exposed weather [3]. This treatment method not only causes serious resource waste but also causes serious damage to soil structure. Recycled permeable concrete (RPC) refers to the permeable concrete made by secondary processing of construction waste, using the processed construction waste as aggregate for concrete preparation, combined with water and cement [4]. The emergence of this concrete material not only solves the problem of handling construction waste but also alleviates the supply and demand of natural aggregates [5]. RPC’s unique honeycomb-like structure can play a good role in noise reduction, water absorption, and suction. Using RPC as the main material for urban road surfaces can effectively alleviate the urban heat island effect [6]. However, the mechanical properties of RPC itself limit its further development and growth. According to scholars’ research, the three main factors affecting RPC strength and permeability are aggregate particle grading (APG), target porosity (TP), and water-cement ratio (WCR). Therefore, in this study, the three factors mentioned above are used as variables, and significance analysis is used to analyze the impact of each factor on RPC. Based on the influence level of different factors, the material ratio of RPC is optimized and studied. The purpose is to improve the mechanical properties of RPC to meet the needs of urban construction.

The innovation of the research lies in the comprehensive consideration of the influence of WCR, TP, and APG distribution on the performance of reclaimed permeable concrete, and the optimization of concrete ratio by volume method. The study provides a novel multivariate interaction analysis method for a deeper understanding of how these factors collectively affect the performance of concrete. Moreover, it emphasizes the importance of precise control of aggregate particle size distribution (PSD) in regulating the pore structure, permeability, and strength of concrete. In addition, the study also discusses the economy and market application prospects of recycled aggregate, thereby providing a scientific foundation and market guidance for the utilization of green and low-carbon building materials.

The unique contribution of this study lies in the comprehensive consideration of the effects of water cement ratio, target porosity and aggregate particle size distribution on the performance of recycled permeable concrete, and the optimization of concrete ratio. By optimizing the concrete ratio by volume method, this study provides a multivariable interactive analysis method to deepen the understanding that these factors jointly affect the concrete performance.

This study innovatively utilizes significant analysis to clarify the influence of different factors on the mechanical properties of concrete, and optimizes the concrete mix ratio. This study is conducted from four aspects. Firstly, a review is conducted on the current research status of RPC in different countries. Secondly, the structural optimization application of green low-carbon concrete technology is emphasized. Thirdly, the performance of RPC is analyzed and a summary of the research content is performed.

## 2. Related works

RPC is an excellent green and low-carbon economic material. X. Cai et al. found that the high water absorption of recycled aggregate concrete (RAC) can lead to mud leakage in structural design. In response to this issue, the author used the optimization volume method to optimize the mixture ratio of RPC and conducted corresponding material compressive strength (CS) experiments. Concrete containing 15% carbon black in the material was suitable for roads with medium to low traffic volume, while concrete without carbon black was suitable for roads with high traffic volume [7]. A. M. Aragoncillo et al. proposed a resistivity testing method to investigate the permeability of RAC and measured the permeability and rapid chloride ion permeability of RPC. When the WRC was low, the permeability of permeable concrete was lower [8]. B. Ali believed that replacing natural aggregates with recycled aggregates can cause damage to the ductility of concrete materials. Therefore, the author proposed using hybrid fibers to enhance the ductility. The combination of polypropylene fibers, hooked steel fibers, and hybrid fibers could effectively improve the ductility of RPC [9]. S. Jian et al. proposed using granulated blast furnace slag instead of partially recycled aggregate to improve the wear resistance of RPC. After adding slag and reducing the amount of cement, the wear resistance of RPC increased by 38.78%, and the porosity and permeability of concrete also increased accordingly [10].

The use of RPC is becoming increasingly widespread. H. Luan et al. analyzed the appearance, loss, dynamic elastic modulus, and strength loss of RPC after freeze-thaw cycles to test its freeze-thaw resistance. The doping in recycled aggregates would directly affect the freeze-thaw resistance of RPC [11]. A. B. Malayali et al. replaced natural aggregates with recycled brick aggregates (RBA) to investigate the role of recycled aggregates in geopolymer perspective concrete. Furthermore, RPC was prepared using NaOH and sodium silicate solutions as activators, and its durability and CS were analyzed. The results showed that adding RBA to concrete could reduce the strength performance of the component [12]. K. Liu et al. found that residual mortar in concrete could form weak interface transition zones, which has an impact on the performance of recycled concrete. In response to this issue, the author proposed a carbonization treatment method to treat recycled concrete. The proposed method could effectively improve the water absorption of recycled concrete and enhance the weak interface transition zone of concrete [13]. S. Jagan et al. proposed to improve the performance of recycled concrete by using different mixing methods to enhance the quality of concrete. The author proposed four methods: two-stage mixing method, mortar mixing method, sand wrapping mixing method, and double mixing method. Among them, the concrete prepared by method two had better strength performance [14]. The two-stage mixing method is predominantly employed to regulate the uniformity and functionality of concrete. The double mixing method is principally focused on enhancing the uniformity of concrete and regulating the temperature of mass concrete. D. Sun et al. found that recycled concrete has the characteristics of high porosity and multiple interface transition zones, leading to harmful ions easily entering the concrete and reducing its strength performance. Given this, the author proposed to increase the viscosity of mortar and conducted mud soaking operations to improve the performance of concrete. This method has been proven to be effective in increasing the strength of concrete [15]. Çelik et al. proposed experimental plans for replacing coarse and fine-aggregates with different proportions of ground glass powder and crushed glass as substitutes for aggregates in concrete. The results showed that waste glass powder enhanced the volcanic ash effect and improved the strength of concrete. Especially when used as a substitute for fine-aggregates, the tensile strength increased by 14% and the bending strength increased by 3.2% -11.1%, respectively. However, replacing coarse aggregates with large particle glass led to a decrease in strength [16]. Martínez García et al. proposed using waste wood ash as a partial alternative material for the production of geopolymer concrete and mortar in response to the demand for cement substitute materials. The results showed that waste wood ash could effectively serve as a filling material, and adding it in moderation could provide high CS, which helped reduce industrial waste and environmental pollution [17]. Gyawali proposed a solution for the management of concrete/brick fragments after the 2015 Nepal earthquake, which involves recycling these materials as coarse and fine aggregates and powders. The results showed that the recovered fine-aggregate reduced the slump and slightly reduced the strength, while the recovered powder significantly reduced the strength [18].

In summary, RPC is a common green and low-carbon building material in the construction industry. However, compared to natural aggregate concrete, it has the problem of lower structural strength. As a building material, this defect is undoubtedly fatal. Therefore, many scholars have conducted study on the performance of RPC in terms of the type or content of recycled aggregates. As for the relationship between porosity and structural strength, this study takes this as a direction to explore the relationship between the two, and optimizes the ratio of RPC.

## 3. Materials and methods

RPC is a common green low-carbon economic material in the construction industry. This chapter will explore the structural optimization of RPC from two aspects. Firstly, it is an analysis of the factors that affect the CS of RPC, and secondly, an optimization study of the RPC admixture ratio.

### 3.1 Significance analysis of factors influencing RPC compressive strength

**Fig 1:**
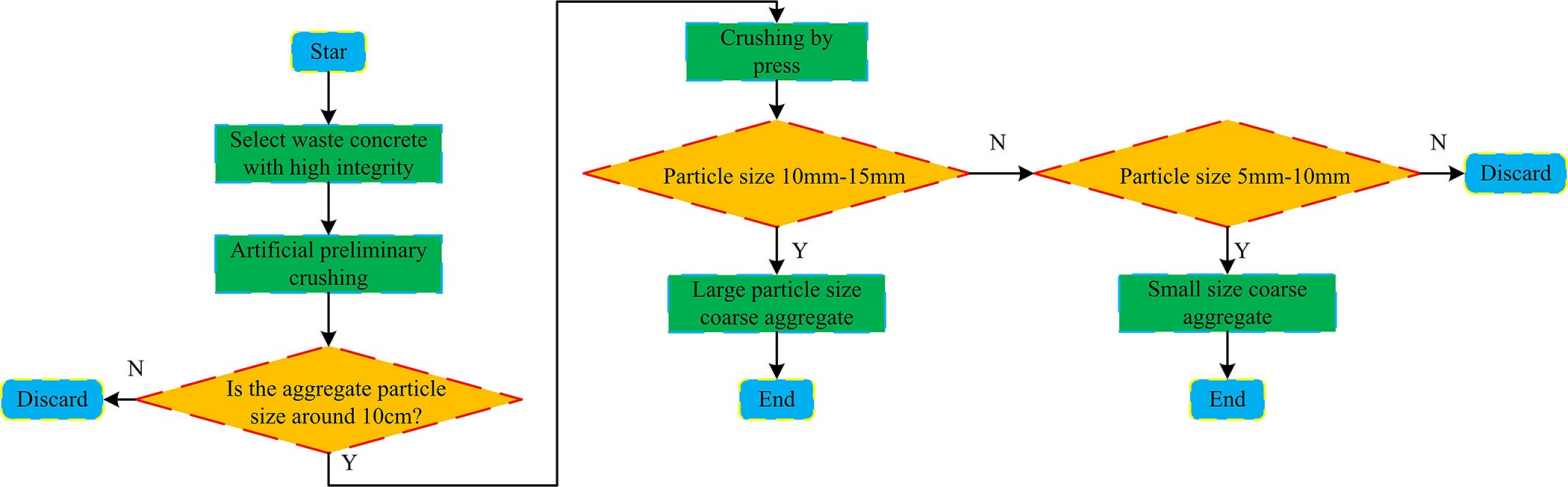
Production process of coarse aggregate.

The mechanical properties of RPC are the main factor affecting its usage. However, the current common research on RPC performance is still limited to variables such as concrete WCR, aggregate content, and aggregate particle size, with limited research on porosity and APG. This study will investigate the mechanical properties of RPC from WCR, APG, and TP. The recycled aggregates used in the experiment are all from waste concrete generated during house renovation and demolition. The preparation of recycled aggregate first selects relatively complete waste concrete as the raw material and undergoes manual preliminary crushing (the particle size of the aggregate obtained after manual crushing is about 10cm). Secondly, a press is used to process the crushed aggregate obtained in the previous step into an aggregate with a particle size of approximately 15mm. Finally, after manual screening, coarse aggregates with different particle sizes can be obtained. Fig 1 shows the specific flow of coarse aggregate.

**Fig 2:**
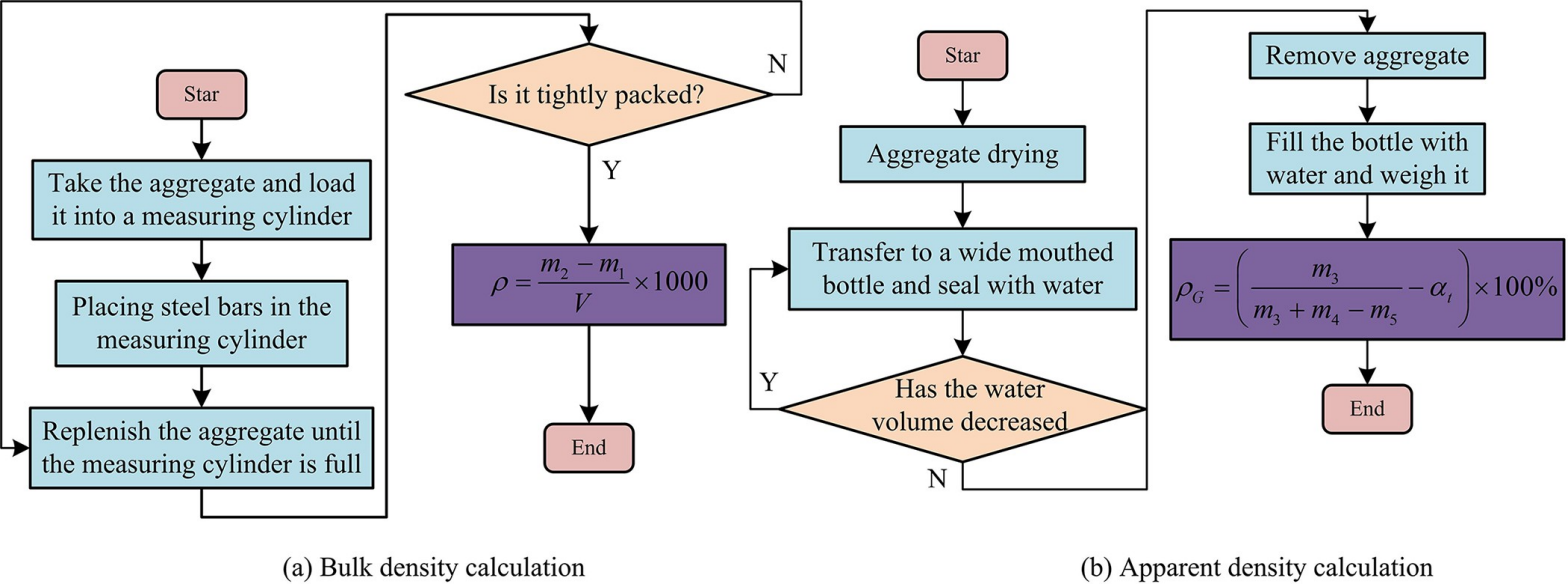
Calculation process of aggregate BD and AD.

From Fig 1, recycled aggregates will produce aggregates that do not meet experimental requirements during the crushing process. To reduce the experimental cost, these aggregates that do not meet the requirements of the experiment will not be secondary crushing. The experiment will be screened directly in the initially broken recycled aggregate. If the recycled aggregate that meets the requirements is insufficient, the recycled aggregate that does not meet the requirements should be broken and screened twice. The coarse aggregates selected for the experiment are divided into two levels: large and small. The size of small aggregates ranges from 5mm to 10mm, while the large size aggregates ranges from 10-15mm. The experiment also requires calculating the bulk density (BD), apparent density (AD), water absorption (WA), and crushing index (CI) of the aggregate. The calculation process of BD is as follows: Take a sample and load it into a measuring cylinder, and fill it in two layers. After the first layer is filled, a steel bar with a diameter of 1 cm needs to be placed at the bottom of the measuring cylinder, and the aggregate needs to be compacted before filling the second layer of aggregate. After the second layer is filled, it will continue to be compacted. Aggregate will be added to the measuring cylinder, scraped flat along the mouth of the cylinder, and weighed. Then, the calculation is performed using Formula (1) [19], as shown in Fig 2A.


$$\rho =\frac{m_{2}-m_{1}}{V}\times 1000$$ (1)

In Formula (1), *ρ* represents BD (kg/m^3^). *m*_1_ represents the mass of the measuring cylinder (g). *m*_2_ represents the gross of the measuring cylinder and experimental aggregate (g). *V* represents the volume of the measuring cylinder (m^3^). The calculation process for aggregate AD is as follows: Take a certain quality of dried aggregate, put it into a wide mouthed bottle and inject water (the bubbles in the bottle need to be eliminated during injection), and seal for 24 hours. Afterwards, continue to inject water and seal for 24 hours until the water content in the sealed bottle remains unchanged. Weigh the sealed bottle with aggregates. Afterwards, remove the aggregate, fill the bottle with water, and weigh it again. Formula (2) can be used to calculate aggregate AD, as shown in Fig 2B [20].


$$\rho_{G}=(\frac{m_{3}}{m_{3}+m_{4}-m_{5}}-\alpha_{t})\times 100\%$$ (2)

In Formula (2), *ρ*_*G*_ represents AD (kg/m3). *m*_3_ represents the dry density mass of the sample (MoS, g). *m*_5_ represents the total MoS, water, and bottle (g). *m*_4_ represents the total weight of water and bottle (g). *α*_*t*_ represents the correction coefficient for the effect of water temperature on AD.

**Fig 3:**
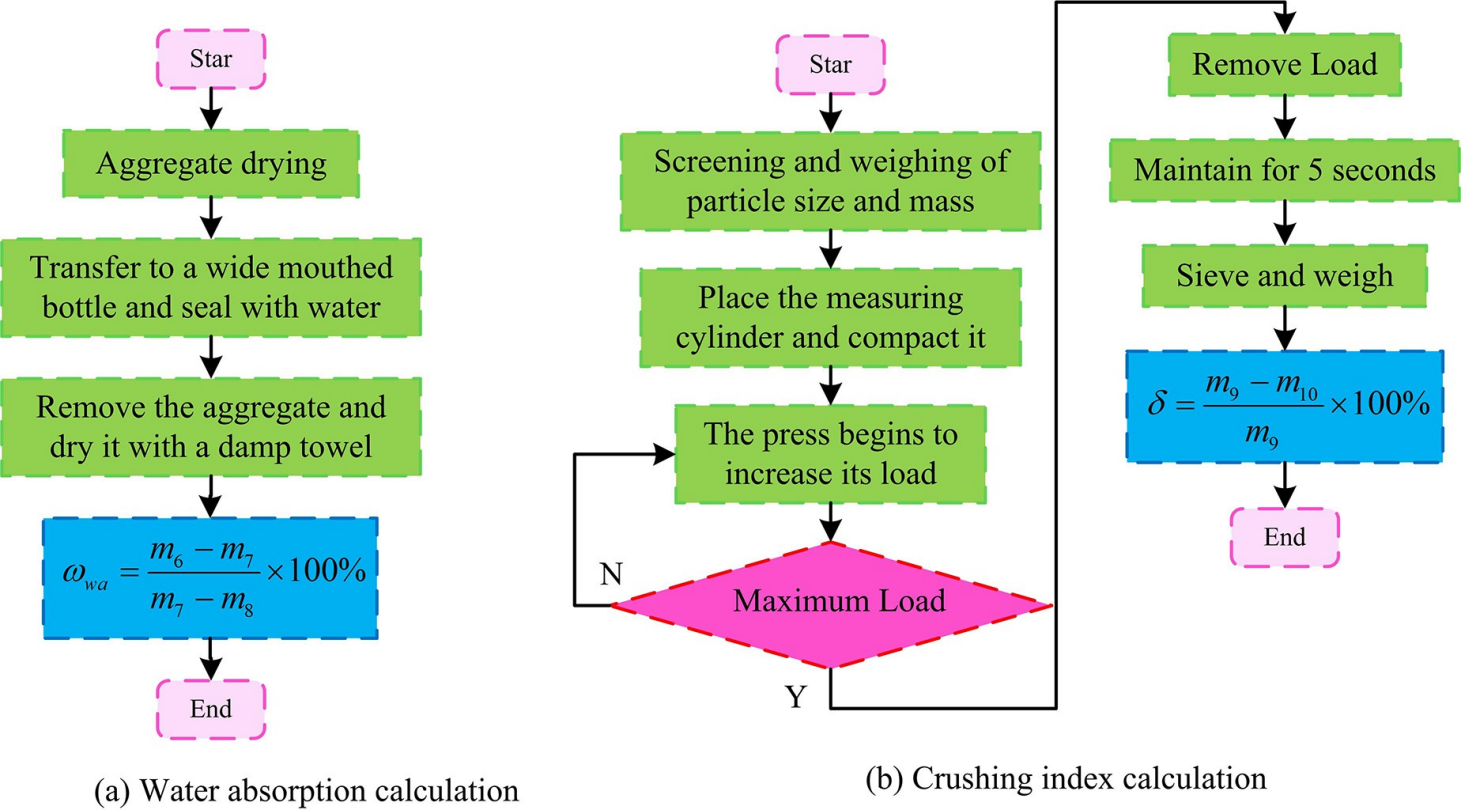
Calculation process for WA and CI.

The calculation process of WA: After drying and cooling a portion of the aggregate, weigh its mass. Place the weighed aggregate into a wide mouthed bottle and fill it with water, remove any bubbles, and seal for 24 hours. Take out the aggregate and dry it with a wet towel before weighing it. The mass is recorded and calculating it using Formula (3), as shown in Fig 3A [21].


$$\omega_{wa}=\frac{m_{6}-m_{7}}{m_{7}-m_{8}}\times 100\%$$ (3)

In Formula (3), *ω*_*wa*_ represents WA (%). *m*_7_ represents the quality of the sample and tray after drying. *m*6 represents the quality of the sample and tray before drying. *m*8 represents the quality of the pallet. Calculation of CI: Use screens of different scales to separate the mass of large and small sized aggregates, place the aggregates into a measuring cylinder, and compact them. Place the measuring cylinder into the press for load loading, reach the maximum load and maintain it for 5 seconds, then unload. The loaded aggregate is taken out for screening and weighing, and calculating the aggregate CI using Formula (4), as shown in Fig 3B.


$$\delta =\frac{m_{9}-m_{10}}{m_{9}}\times 100\%$$ (4)

In Formula (4), *δ* represents CI. *m*_9_ represents the MoS. *m*_10_ represents the MoS that has not been sieved after crushing.

The analysis of concrete performance will be carried out in four directions: CS experiment, flexural strength (FS) experiment, actual porosity test of concrete, and permeability test. The CS experiment is conducted using a fully automatic pressure testing machine: the prepared and cured test blocks are taken out, placed flat in the middle of the pressure testing machine, and uniformly pressed at a speed of 0.2MPa/s. The pressure value of the test blocks is calculated, and then the standard CS value of the test blocks is calculated using Formula (5).


$$f_{cc}=\frac{F}{A}•\alpha$$ (5)

In Formula (5), *f*_*cc*_ represents the standard CS (MPa) of the test block. *F* represents the failure load of the test block (N). *A* represents the compressive bearing area of time (m^2^). *α* represents the correction factor. The experimental environment for FS and CS is consistent, but the loading speed for FS experiments is 200N/S. The standard FS calculation formula for the test block is shown in Formula (6) [22].


$$F_{f}=\frac{PL}{bh^{2}}•\alpha$$ (6)

In Formula (6), *F*_*f*_ represents the FS (MPa) of the test block. *L* represents the spacing between supports (m). *b* represents the width of the specimen interface (m). *h* represents the cross-sectional height of the specimen (m).The formation of pores is caused by the hydration and heat release of cement during the pouring process of concrete. Concrete pores include fully enclosed pores (FEP), semi-enclosed pores (SEP), and through pores. Among them, FEP is an invalid pore that has little impact on the specimen. The testing of specimen porosity is to detect the effective pores, namely SEP and through pores, of the specimen. The commonly used porosity testing methods currently include weighing method, density method, and gas volume method. The weighing method has the characteristic of being simple and easy to implement. Therefore, this study uses this method to test the actual porosity of concrete. To measure the porosity of a specimen using the weighing method, it is necessary to obtain the weight of the specimen in both saturated and dry states, and then calculate the actual porosity of the specimen according to Formula (7).


$$AP=(1-\frac{m_{11}-m_{12}}{V_{s}})\times 100\%$$ (7)

**Fig 4:**
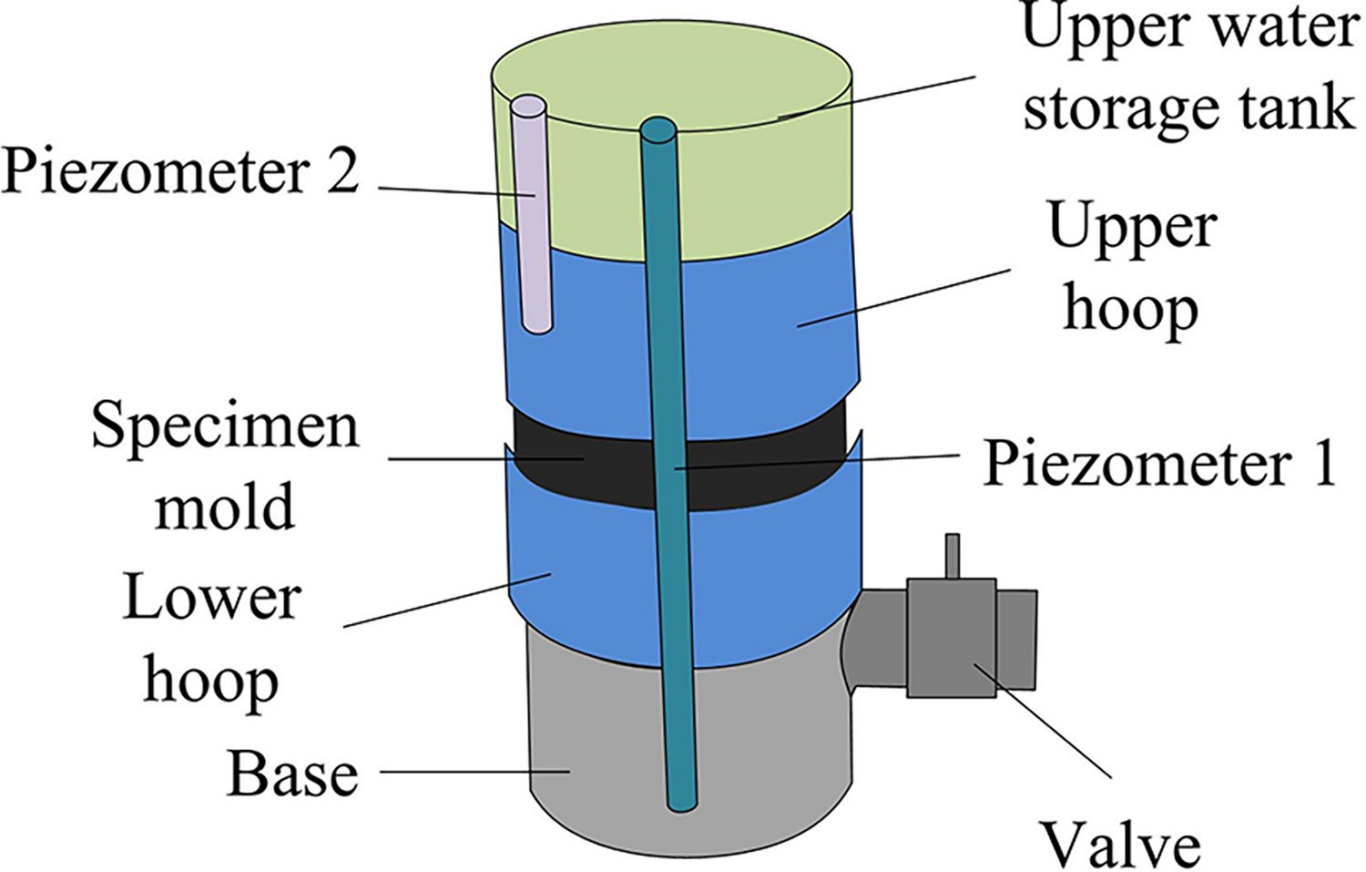
Head loss tester.

In Formula (7), *AP* represents the actual porosity of the specimen. *m*_11_ represents the mass of the specimen in a saturated state. *m*_12_ represents the mass of the specimen in a dry state. *V*_*s*_ represents the volume of the specimen. The permeability of concrete is tested using a self-made head loss tester, as shown in Fig 4.

During the experiment, it is necessary to ensure that the water flow in the head loss instrument is at full flow while maintaining a constant flow rate and velocity. The water level in the upper storage tank of the tester also needs to be maintained at the highest position to provide a constant water head for the tester. After the water level of the tester stabilizes, it is necessary to record the water level heights in the two pressure measuring pipes. The difference between the two water level heights is the head loss of the specimen. The expenses on the valve of the tester are maximized, and water is continuously injected while ensuring smooth water flow, ensuring that the water level in the upper storage tank is always maintained at the highest level. After the water flowing out of the tester stabilizes, a measuring cylinder is used to measure the water flow rate within 10 seconds, and then the permeability coefficient (PC) *K* of the specimen is calculated using Formula (8).


$$K=\frac{V_{l}\times L_{1}}{t\times A\times \Delta h_{1}}$$ (8)

In Formula (8), *V*_*l*_ represents the water flow rate. *L*_1_ represents the length of the pattern. *t* represents the measurement time. *A* represents the cross-sectional area of the pattern. Δ*h*_1_ represents the head difference.

### 3.2 Optimization design of RPC mix ratio based on volume method

**Fig 5:**
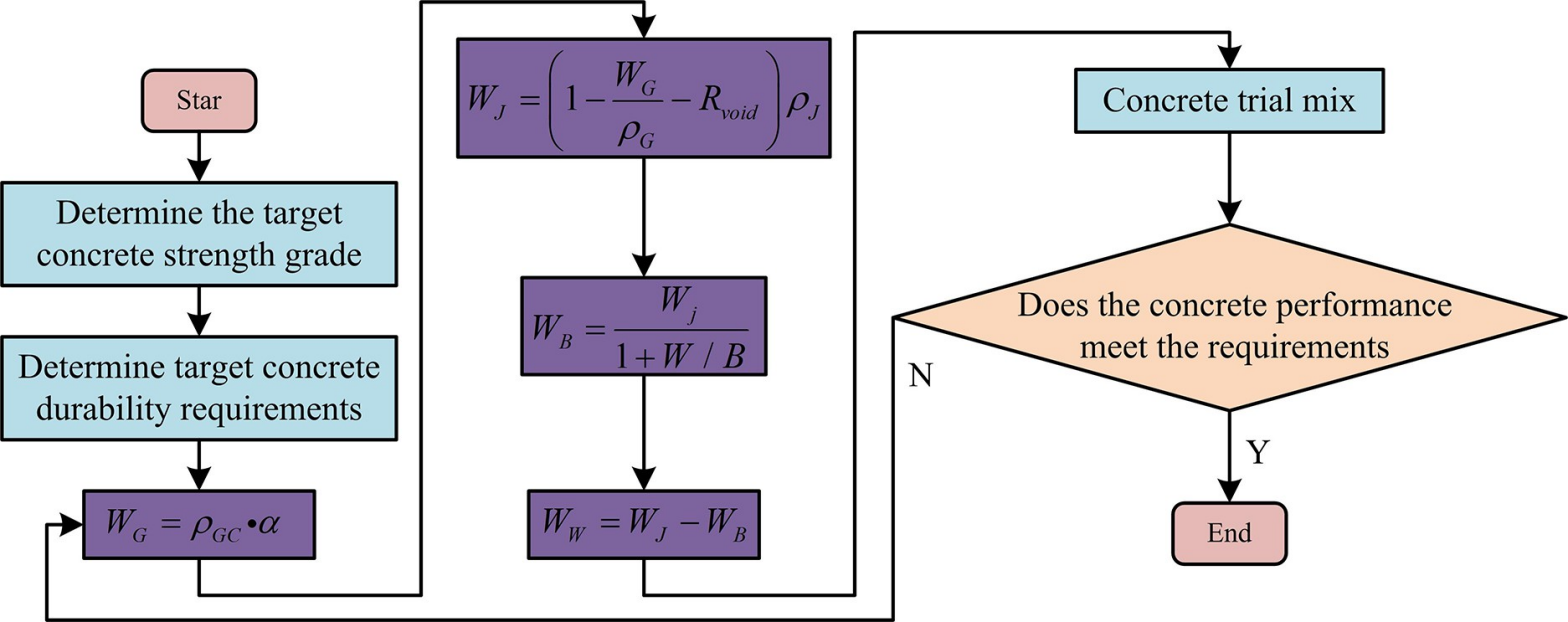
Design process for the RPC proportion.

Common concrete mix design methods include volume method, mass method, and specific surface area method. Different mix design will have different effects on the basic mechanical properties of concrete. When designing concrete mix proportions using the volumetric method, it has the characteristics of high accuracy and comprehensive consideration. Therefore, this study focuses on the volume method to optimize the concrete mix ratio. Fig 5 shows the process of preparing RPC.

Permeable concrete is mainly composed of coarse aggregate and does not use fine aggregate, therefore it has a non-dense frame structure. Its main materials include coarse aggregate, cementitious slurry, and additives that reduce water content and thickening agents. The proportion design of permeable concrete is different from that of ordinary concrete. While considering the influence of concrete strength, the aggregate gradation and physical compactness also have an impact on the mix design method. When using the volume method to prepare RPC, it is necessary to calculate the amount of aggregates, water, binder paste, cement, and admixtures per unit volume of concrete (Con/vol). The dosage of additives needs to be determined based on the mixing results, and other materials need to be obtained through calculation. The calculation of coarse aggregate dosage is Formula (9).


$$W_{G}=\rho_{GC}•\alpha$$ (9)

In Formula (9), *W*_*G*_ represents the quantity of coarse aggregate used Con/vol. *ρ*_*GC*_ represents the compact BD of coarse aggregate (kg/m^3^). The calculation of adhesive dosage is Formula (10).


$$W_{J}=(1-\frac{W_{G}}{\rho_{G}}-R_{void})\rho_{J}$$ (10)

In Formula (10), *W*_*J*_ represents the amount of binder Con/vol (kg/m^3^). *ρ*_*G*_ represents the AD of coarse aggregate (kg/m^3^). *R*_*void*_ represents the design value of porosity. *ρ*_*J*_ represents the density of the cementitious slurry when the WCR changes (kg/m^3^). The calculation of cement dosage is Formula (11).


$$W_{B}=\frac{W_{j}}{1+W/B}$$ (11)

In Formula (11), *W*_*B*_ represents the cement dosage Con/vol (kg/m^3^). *W*/*B* represents WCR. The calculation of water consumption is Formula (12) [23].


$$W_{W}=W_{J}-W_{B}$$ (12)

In Formula (12), *W*_*W*_ represents the mixing water consumption Con/vol. When designing the material ratio of permeable concrete using the volume method, the filling and wrapping theory is used. Coarse aggregates are kept tightly packed in permeable concrete. The cementitious slurry will fully wrap it while connecting other parts to fix the position of the coarse aggregate in the concrete, stabilizing it and forming the skeleton structure of the concrete. The connection between various materials in concrete relies on the cementitious slurry. After solidification and hardening, the cementitious slurry will form a pore structure. These pores are divided into effective pores and ineffective pores. The volumetric method is based on the unique skeleton structure of permeable concrete. Assuming that concrete aggregates, slurries, and pores together form the same system, the volume of the system is a fixed value. When designing the concrete mix ratio, a TP is set to calculate the amount of coarse aggregate used in this volume of concrete based on the pores in the dense accumulation of coarse aggregate itself. Finally, the use of coarse aggregate and TP can be used to calculate the amount of slurry used. When designing the proportion of permeable concrete using the conventional volume method, the main impact of WCR on the performance of concrete is on it. However, when analyzing the effects of different factors on concrete performance, the coarse APG and porosity on concrete performance influence is also considered. Therefore, this study proposes a permeable concrete mix design method that considers APG and porosity. This method is still based on the volume method. During implementation, it is necessary to obtain equations for actual porosity related to APG and TP, equations for CS related to actual porosity, equations for FS related to actual porosity, and equations for PC related to APG and TP. The equation for actual porosity with respect to APG and TP is shown in Formula (13).


$$AP=0.13+0.89X-1.14Y+0.8Y^{2}$$ (13)

In Formula (13), *X* represents the TP of concrete. *Y* represents APG. The equation for actual porosity in CS can be found in Formula (14).


$$F_{CC}=0.014AP^{2}-1.104AP+30.27$$ (14)

In Formula (14), *F*_*CC*_ represents CS. The equation for actual porosity in FS can be found in Formula (15).


$$F_{f}=0.001AP^{2}-0.129AP+3.772$$ (15)

The equation for the PC with respect to APG and TP is shown in Formula (16).


$$K=-0.52+0.33X-0.22Y+0.01Y^{2}$$ (16)

**Fig 6:**
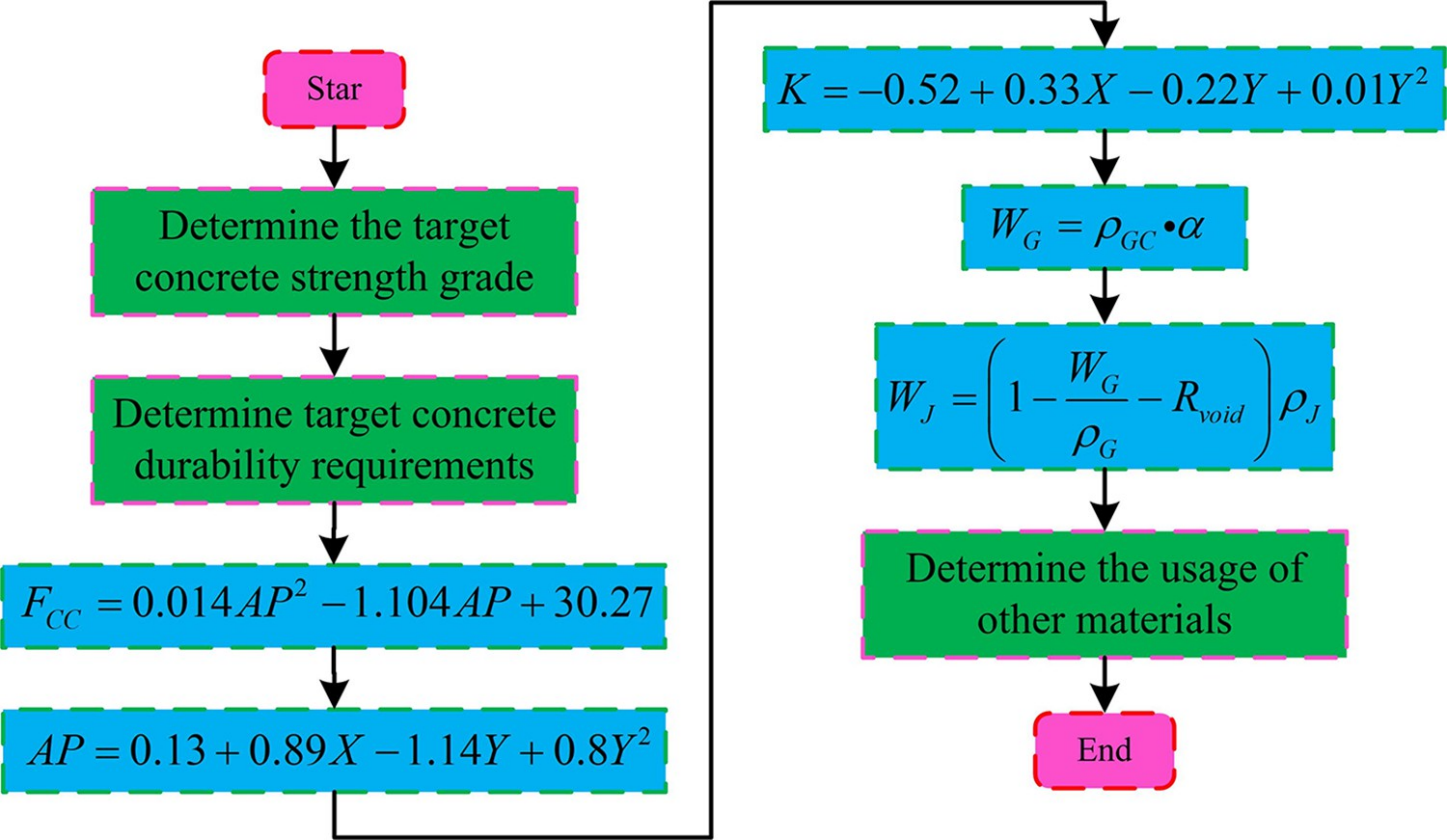
The design process of permeable concrete ratio, considering particle grading and porosity.

The process of the research method: Firstly, based on the performance requirements of permeable concrete, the actual porosity of the concrete is used as a variable, and an appropriate actual porosity is selected according to Formula (14). Step 2 is to select the more excellent TP and APG according to Formula (13). Step 3 is to substitute the TP and APG selected in steps 1 and 2 into Formula (16) to further screen TP and APG that meet the permeability requirements. Step 4 is to measure the density BD and AD of the selected aggregate, and substitute them into Formulas (9) and (10) to calculate the amount of coarse aggregate and the amount of cementitious slurry. Step 5 is to calculate the amount of other materials used, conduct trial mixing of the concrete, and determine the amount of additives based on the above calculation data. Step 6 is to prepare finished permeable concrete. Fig 6 shows the specific process.

When testing sample performance, it is necessary to measure the actual porosity of the target sample. The measurement process must ensure that all concrete samples are prepared according to standardized procedures to reduce errors caused by inconsistent sample preparation. Precision instruments are used to measure the dry density and saturated surface dry density of concrete samples. Dry density is determined by drying the sample at 105°C to a constant weight and measuring its mass and volume. The saturated surface dry density is determined by soaking the sample to saturation, wiping off the surface moisture with a wet towel, and then measuring its mass and volume. The actual porosity of the target sample is 1 minus the ratio of the sample’s dry density to saturation density. To ensure the accuracy of the measurement results, the study conducts porosity tests on each sample at least three times to ensure data reliability and reduce the impact of random errors. All measuring equipment is regularly calibrated and maintained to ensure the accuracy of measurement results.

## 4. Results

Section 2 proposes a design method for the proportion of permeable concrete that considers APG and porosity. Therefore, section 3 conducts experimental verification of this designed method. The section is divided into two subsections. The first subsection analyzes the sensitivity of RPC and the second subsection analyzes the economic effects of RPC.

### 4.1 Sensitivity analysis of RPC permeability coefficient

**Fig 7:**
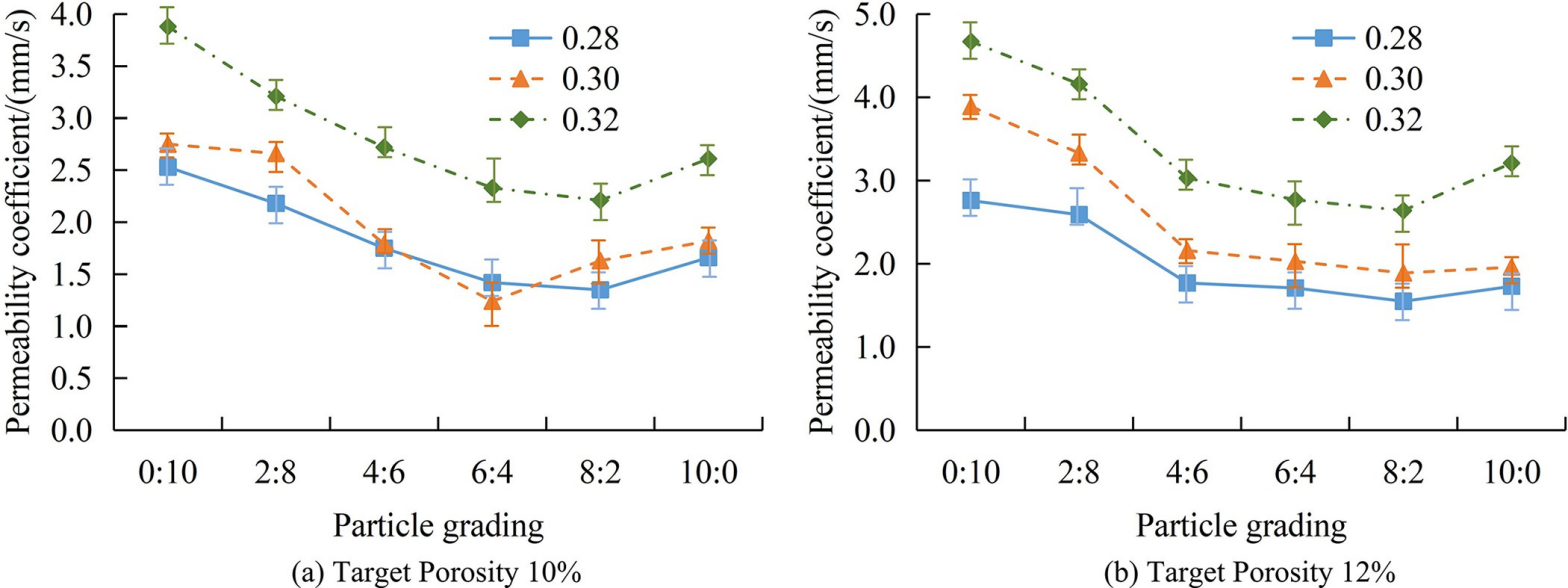
The VoPC and APG of concrete with TP of 10% and 12%.

All data analysis is conducted using laboratory computers. The computer processor version is Intel(R)Core(TM) i5-4460, with CPU@3.20GHz, installed memory of 16.0GB, system type of 64 bit operating system, and experimental data analysis platform of MATLAB. The number of experimental samples in the research is 5 groups. On the premise of ensuring the reproducibility of the experiment, the experimental cost should be reduced as far as possible. During the experiment, to reduce the influence of environmental temperature and other factors on the experimental results, the laboratory temperature is controlled within the interval of 25°C±1°C during the experiment, and other environmental influence factors are also controlled by similar practices. The TP of the experimental sample is divided into 10%, 12%, 14%, and 16%, the WCR is divided into 0.28, 0.30, and 0.32, and the APG is 0:10, 2:8, 4:6, 6:4, 8:2, and 10:0. This study first analyzes the relationship between the PC of concrete and WCR under different TP conditions. The variation pattern between the PC of concrete and APG when TP is 10% and 12% is shown in Fig 7.

**Fig 8:**
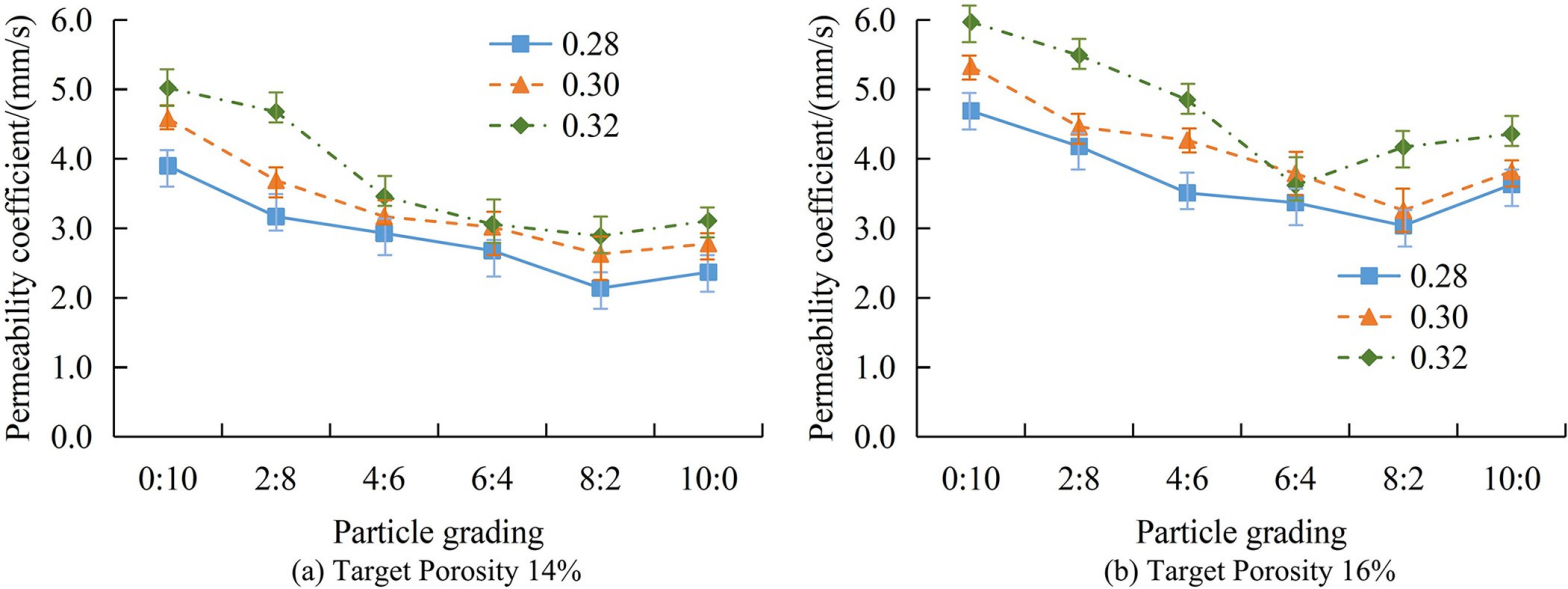
The VoPC and APG of concrete with a TP of 14% and 16%.

Fig 7A shows the variation of permeability coefficient (VoPC) with APG when TP is 10%. Regardless of the WCR, the PC continuously decreases with the increase of APG. When WCR is 0.28 and APG is 0:10, the permeability coefficient of the sample (PCoS) is 2.53mm/s. When the APG is 10:0, the PCoS is 1.66mm/s, a total decrease of 0.87mm/s. When WCR is 0.30 and APG is 0:10, the PCoS is 2.75mm/s. When the APG is 10:0, the PCoS is 1.82mm/s, a total decrease of 0.93mm/s. When WCR is 0.32 and APG is 0:10, the PCoS is 3.88mm/s. When the APG is 10:0, the PCoS is 2.61mm/s, a total decrease of 1.27mm/s. Fig 7B shows the VoPC with APG when TP is 12%. Regardless of the WCR, the PCoS is always the lowest when the APG is 8:2. When the WCR is 0.28, the minimum PC of the pattern is 1.55mm/s. After the APG changes to 10:0, the PCoS increases to 1.73mm/s. When the WCR is 0.30, the minimum PC of the pattern is 1.89mm/s. After the APG changes to 10:0, the PCoS increased to 1.96mm/s. When the WCR is 0.32, the minimum PC of the pattern is 2.64mm/s. After the APG changes to 10:0, the PCoS increases to 3.21mm/s. Good PSD can increase the friction between aggregates, reduce porosity, and thus lower permeability. A lower WCR usually means less cement paste, thereby reducing porosity and improving the compactness of concrete, which reduces permeability. The permeability continues to decrease with the increase of APG, indicating that good aggregate grading can increase the friction between aggregates, reduce porosity, and thus reduce the permeability. The permeability coefficient is the lowest at an APG of 8:2, indicating that the optimal equilibrium point may be reached under a specific APG, minimizing the gap between aggregates and achieving the lowest permeability coefficient. Reducing the permeability coefficient can reduce the infiltration of harmful substances, improve the durability of concrete, and extend the service life of structures. For permeable concrete, moderately reducing the permeability coefficient can make it more suitable for roads and other structures that carry traffic loads, while maintaining a certain degree of permeability. This is beneficial for the infiltration and utilization of rainwater. The variation pattern between the PC of concrete and APG when TP is 14% and 16% is shown in Fig 8.

**Fig 9:**
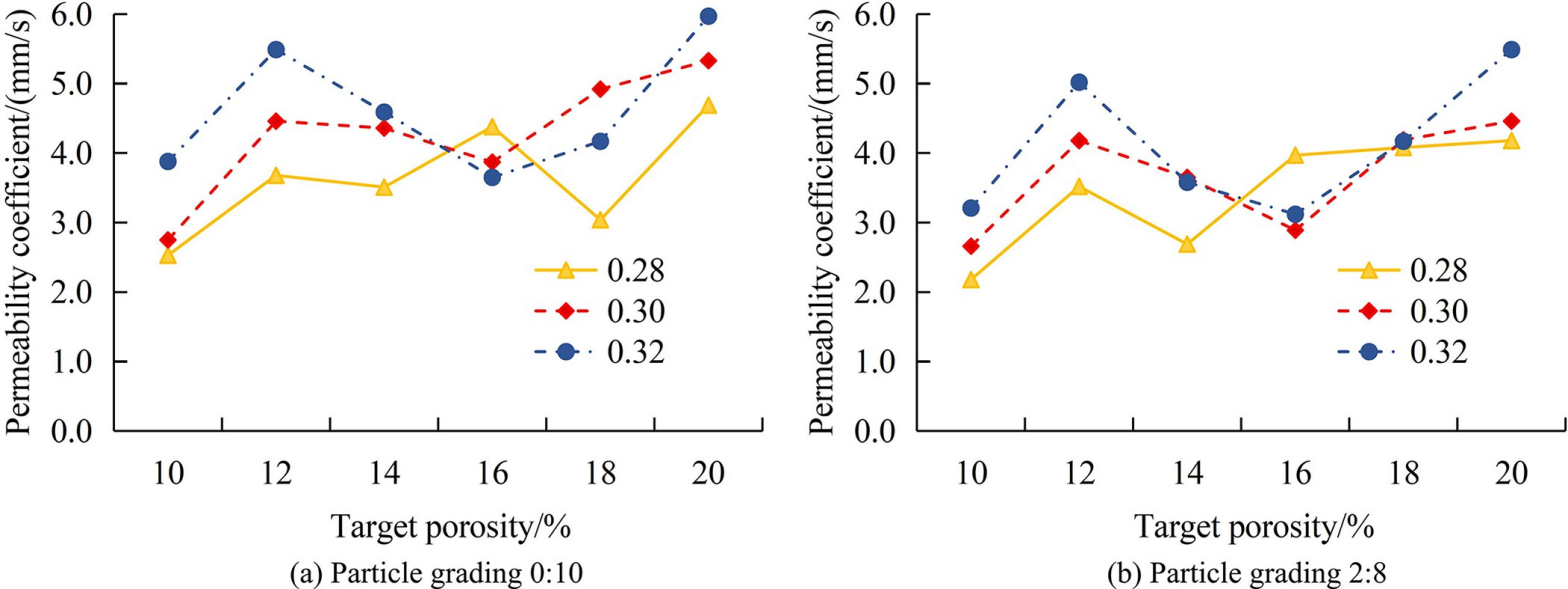
The VoPC with TP when the PSD is 0:10 and 2:8.

Fig 8A shows the VoPC with APG when TP is 14%. Similar to the 12% change in TP, the PCoS continuously decreases during the process of APG changing from 0:10 to 8:2. When it changes from 8:2 to 10:0, the PCoS rebounds. When the WCR is 0.28, the increase in the PCoS is 0.23mm/s, and the overall decrease in the PCoS is 1.53mm/s. When the WCR is 0.30, the increase in the PCoS is 0.15mm/s, and the overall decrease in the PCoS is 1.80mm/s. When the WCR is 0.32, the increase in the PCoS is 0.22mm/s, and the overall decrease in the PCoS is 1.91mm/s. Fig 8B shows the VoPC with APG when TP is 14%. The change in the PCoS is consistent with that when TP is 14%. When the WCR is 0.28, the increase in the PCoS is 0.59mm/s, and the overall decrease in the PCoS is 1.06mm/s. When the WCR is 0.30, the increase in the PCoS is 0.56mm/s, and the overall decrease in the PCoS is 1.51mm/s. When the WCR is 0.32, the increase in the PCoS is 0.19mm/s, and the overall decrease in the PCoS is 1.61mm/s. Compared to the situation where TP is 14%, when TP is 16%, the rebound is higher and the overall decrease is smaller. When the PSD is 8:2, an optimal equilibrium point may be reached, which minimizes the gaps between aggregates and thus achieves the lowest permeability coefficient. The permeability coefficient continuously decreases at APG from 0:10 to 8:2, and rebounds from 8:2 to 10:0. This may indicate that the best locking has the lowest porosity at a specific APG. Compared to TP’s 14%, higher rebound and smaller overall decrease mean that higher porosity makes it more difficult to control the permeability coefficient. By adjusting the PSD and TP, the permeability and strength of concrete can be optimized, thereby improving its structural performance. Appropriate PSD and porosity can ensure sufficient permeability while maintaining the strength and stability of the structure. After optimizing the PSD and porosity, the use of materials can be reduced without sacrificing performance, thereby reducing costs and improving economic benefits. This study also analyzes the VoPC of concrete with TP under different APGs. Fig 9 shows the changes in PSD at 0:10 and 2:8.

**Fig 10:**
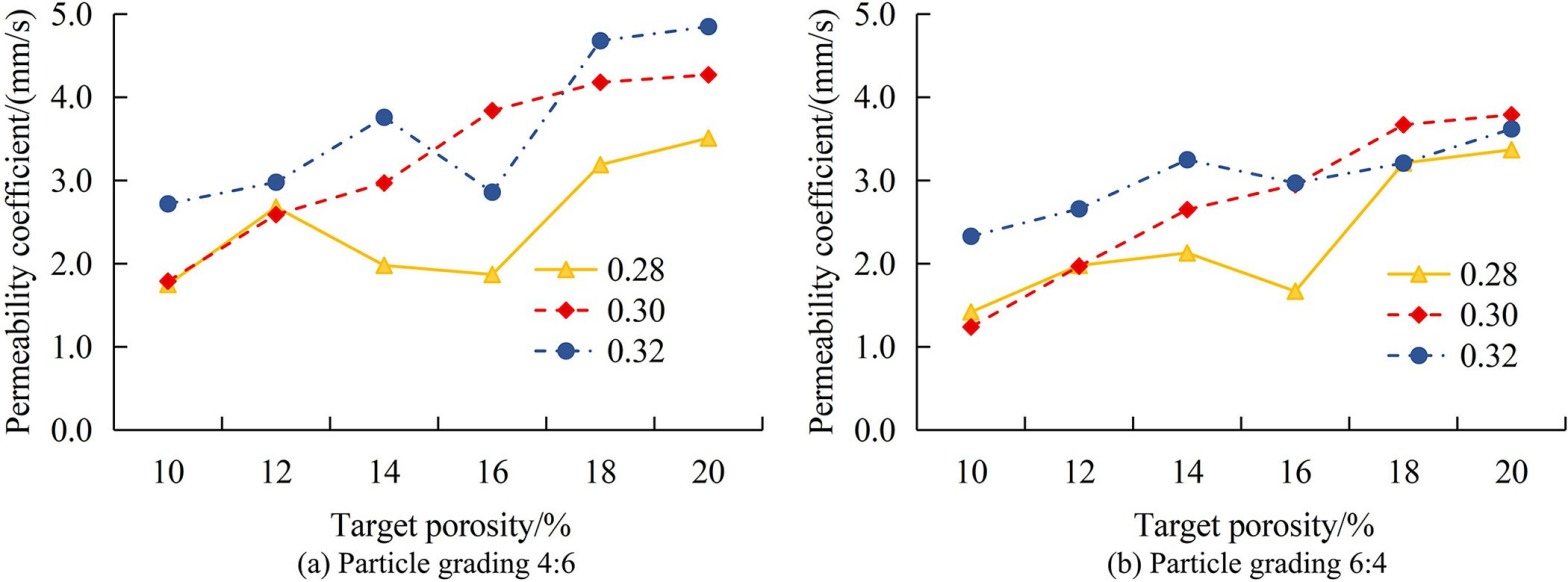
The VoPC with TP when the PSD is 4:6 and 6:4.

Fig 9A shows the VoPC with TP when PSD is 0:10. Regardless of the WCR, as TP increases to 20%, the PCoS also increases. When TP is 10% and WCR is 0.28, the PCoS is 2.53mm/s. When TP is 20%, the PCoS is 4.69mm/s, a total increase of 85.4%. When TP is 10% and WCR is 0.30, the PCoS is 2.75mm/s. When TP is 20%, the PCoS is 5.33mm/s, a total increase of 93.8%. When TP is 10% and WCR is 0.32, the PCoS is 3.88mm/s. When TP is 20%, the PCoS is 5.97mm/s, a total increase of 83.9%. Fig 9B shows the VoPC with TP when PSD is 2:8. When TP is 10% and WCR is 0.28, the PCoS is 2.18mm/s. When TP is 20%, the PCoS is 4.18mm/s, a total increase of 91.7%. When TP is 10% and WCR is 0.30, the PCoS is 2.66mm/s. When TP is 20%, the PCoS is 4.46mm/s, a total increase of 67.7%. When TP is 10% and WCR is 0.32, the PCoS is 3.21mm/s. When TP is 20%, the PCoS is 5.49mm/s, a total increase of 71.0%. The permeability increases with TP, indicating that higher TP results in greater space within the concrete to accommodate moisture, thereby increasing water permeability. The magnitude of the increased permeability exhibits variation due to enhanced locking and reduced pores, signifying a substantial impact of aggregate particle-level pair permeability. As the TP increases, the total amount of pores inside the concrete increases, providing more water permeation paths and thus increasing the permeability coefficient. When the PSD is 0:10, the gaps between aggregates may be larger, resulting in higher permeability. When the PSD is 2:8, the increase in permeability coefficient varies due to better interlocking between aggregates and fewer pores. By adjusting the TP, the permeability of concrete can be customized to meet different application requirements, such as roads, drainage systems, etc. It can reduce the use of materials while meeting performance requirements, improve construction efficiency, and thus reduce costs. The changes in the PCoS when the PSD is 4:6 and 6:4 are displayed in Fig 10.

**Fig 11:**
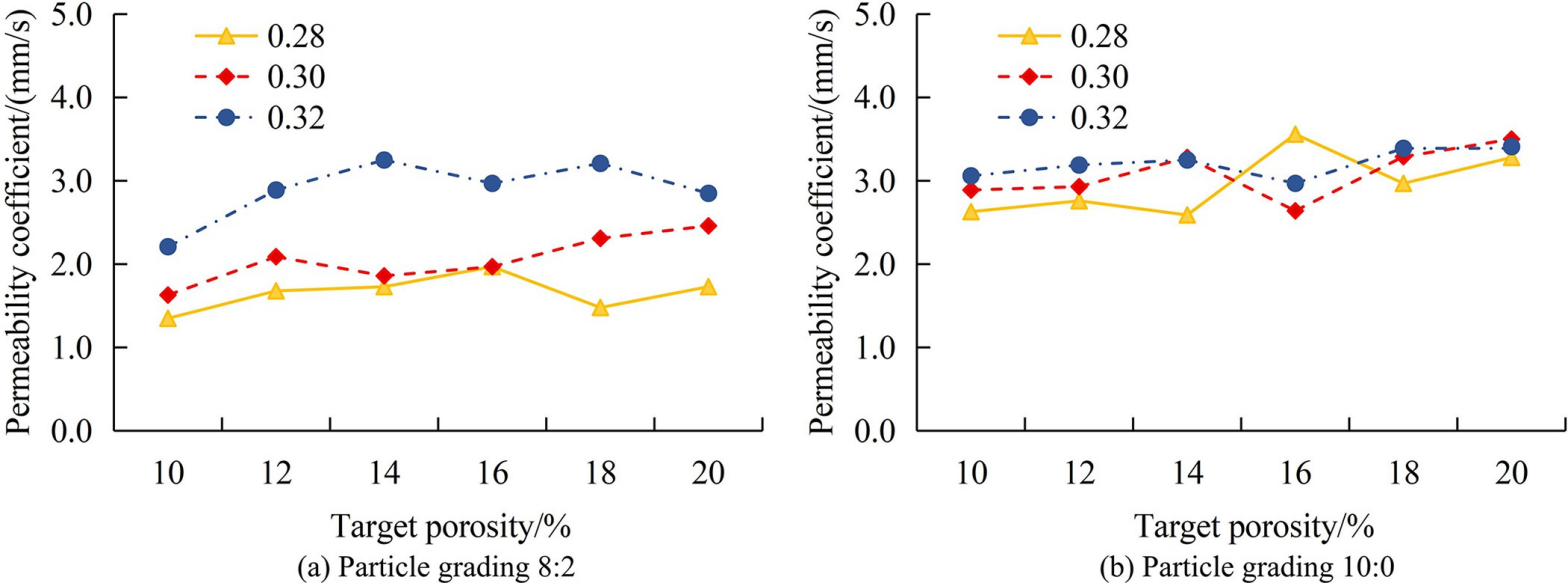
The VoPC with TP when the PSD is 8:2 and 10:0.

Fig 10A shows the VoPC with TP when PSD is 4:6. As the porosity increases, the overall PCoS increases. When TP is 10% and WCR is 0.28, the PCoS is 1.75mm/s. When TP is 20%, the PCoS is 3.51mm/s, a total increase of 100.6%. When TP is 10% and WCR is 0.30, the PCoS is 1.79mm/s. When TP is 20%, the PCoS is 4.27mm/s, a total increase of 138.5%. When TP is 10% and WCR is 0.32, the PCoS is 2.72mm/s. When TP is 20%, the PCoS is 4.85mm/s, a total increase of 78.3%. Fig 10B shows the VoPC with TP when PSD is 6:4. When TP is 10% and WCR is 0.28, the PCoS is 1.42mm/s. When TP is 20%, the PCoS is 3.37mm/s, a total increase of 137.3%. When TP is 10% and WCR is 0.30, the PCoS is 1.24mm/s. When TP is 20%, the PCoS is 3.79mm/s, a total increase of 205.6%. When TP is 10% and WCR is 0.32, the PCoS is 2.33mm/s. When TP is 20%, the PCoS is 3.62mm/s, a total increase of 55.4%. When the WCR is 0.30, the increase in the PCoS is the largest. When the PSD is 4:6 and 6:4, the ratio of large particles to small particles in concrete is different, which affects the BD and pore structure of the aggregate. The water permeability increases with TP, indicating a significant effect on the compactness and pore structure of aggregate size-paired concrete. The water permeability coefficient increases significantly with increasing TP, which may be related to a higher proportion of large particles, resulting in increased porosity. A better PSD can reduce pores and improve the compactness of concrete, but when the porosity increases, even a good PSD is difficult to completely prevent the increase in permeability coefficient. The changes in the PCoS at 8:2 and 10:0 PSD are shown in Fig 11.

Fig 11A shows the VoPC with TP when PSD is 8:2. With the increase of TP, the overall PC of concrete still maintains an increase, but the increase is significantly reduced. When the WCR is 0.28 and the porosity is 10%, the PC is 1.35mm/s. When the porosity is 20%, the PC is 1.73mm/s, and the increase in PC is 28.1%. When the WCR is 0.30 and the porosity is 10%, the PC is 1.63mm/s. When the porosity is 20%, the PC is 2.46mm/s, and the increase in PC is 50.9%. When the WCR is 0.32 and the porosity is 10%, the PC is 2.21mm/s. When the porosity is 20%, the PC is 2.85mm/s, and the increase in PC is 29.0%. The increased porosity means that more space inside the concrete is occupied by the pores. These pores provide more permeability paths for water, thus increasing the permeability coefficient. At a porosity of 10%, there are less pores inside the concrete, and when the porosity increases to 20%, the internal pores increase, providing more water channels. As the porosity increases, the pore size and connectivity may also change. Larger pores and better connectivity can reduce the drag of water molecules through the concrete, thus increasing the permeability coefficient. Fig 11B shows the VoPC with TP when PSD is 10:0. As the porosity increases, the PC decreases, but overall it still maintains an increase. When the WCR is 0.28 and the porosity is 10%, the PC is 2.63mm/s. When the porosity is 20%, the PC is 3.28mm/s, and the increase in PC is 24.7%. When the WCR is 0.30 and the porosity is 10%, the PC is 2.89mm/s. When the porosity is 20%, the PC is 3.50mm/s, and the increase in PC is 21.1%. When the WCR is 0.32 and the porosity is 10%, the PC is 3.06mm/s. When the porosity is 20%, the PC is 3.41mm/s, and the increase in PC is 11.4%. When the PSD is 8:2 and 10:0, the ratio of large particles to small particles in concrete is significantly different, which affects the BD and pore structure of the aggregate. When the PSD is 8:2, there may be a good interlocking between aggregates, reducing porosity and resulting in a smaller increase in permeability coefficient as porosity increases. When the PSD is 10:0, due to the fact that all particles are large, there may be more large pores, resulting in a significant increase in permeability coefficient. Based on the variation of RPC’s PC with APG and porosity, as shown in Table 1, this study draws a visual analysis table for RPC’s PC.

**Table 1:** Visualization analysis of permeability coefficient of aggregate RPC.

Sample number	PC (mm/s)	APG	TP	WCR
1	2.31	1	1	1
2	3.18	1	2	2
3	4.36	1	3	3
4	2.54	2	1	1
5	2.96	2	2	2
6	4.20	2	3	3
7	2.22	3	1	2
8	3.74	3	2	3
9	2.11	3	3	1
10	2.49	4	1	3
11	1.48	4	2	1
12	2.48	4	3	2
13	2.19	5	1	2
14	3.56	5	2	3
15	1.87	5	3	1
16	3.76	6	1	3
17	1.99	6	2	1
18	2.78	6	3	2

**Note:** APG Level 1: 0:10; Level 2: 2:8; Level 3: 4:6; Level 4: 6:4; Level 5: 8:2; Level 6: 10:0. TP level 1: 10%; Level 2: 12%; Level 3: 14%. WCR level 1: 0.28; Level 2: 0.30; Level 3: 0.32.

**Fig 12:**
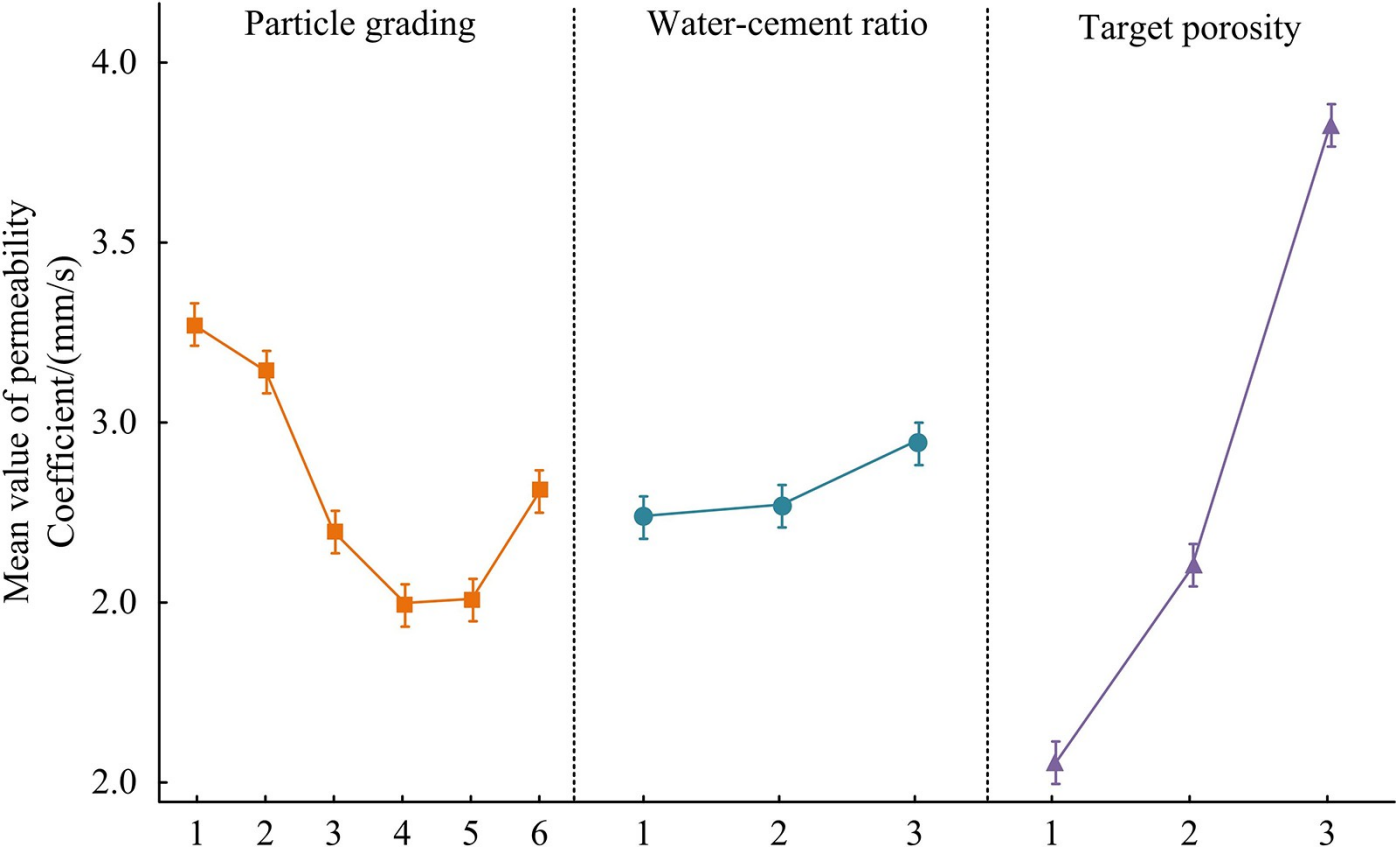
Mean main effect result of PC.

In Table 1, when the APG level is 1–2, the PCoS will increase with the increase of TP and WCR. When the APG is above level 3, the PCoS will decrease with the increase of WCR and TP. The PC of test sample 3 is the highest, at 4.36mm/s. The APG of this sample is level 1, TP is level 3, and WCR is level 3. The PCoS 11 is the lowest, at 1.48mm/s. The APG of this sample is level 4, TP is level 2, and WCR is level 2. The PSD affects the BD and pore structure of aggregates. Different particle gradations can lead to different porosity and pore size distributions, thereby affecting permeability. The TP is directly related to the permeability of concrete. A higher TP means that there is more space in the concrete to accommodate moisture, thereby increasing permeability. The WCR affects the consistency and compactness of cement slurry after hardening. A higher WCR may lead to more pores, as excess moisture leaves more pores during the hardening process of the cement slurry. The main effect results of the mean PC are shown in Fig 12.

In Fig 12, as the APG level increases, the average PC shows a trend of first decreasing and then increasing. When the APG level is 4, the average PC decreases to the lowest, and at this time, the average PC is 2.50mm/s (The difference is statistically significant, *p*<0.01). As the WCR level increases, the average PC shows a gradual upward trend. During the process of increasing from level 1 to level 3, the average PC increases by 0.20mm/s (The difference is statistically significant, *p*<0.05). With the increase of TP, the PC significantly increases, from level 1 to level 3, with an average increase of 1.78mm/s (The difference is statistically significant, *p*<0.001). By adjusting the PSD, WCR, and TP, the permeability of concrete can be effectively controlled. When designing and constructing concrete structures with specific performance requirements, the optimal balance between structural performance, durability, and economic benefits can be achieved. A larger aggregate particle size can better fill the space and reduce pore formation. However, if the large-size aggregate is too abundant, it may lead to the thinning of the cement slurry layer between the aggregates, thus increasing the porosity. Good grading can improve the aggregate packing density and reduce the porosity. Conversely, poor grading may result in large inter-spaces between the aggregates and increased porosity. PSD affects the pore connectivity. Appropriate particle size combination can form more closed pores and reduce water permeability, while inappropriate particle size combination may lead to more open pores and increase water permeability. Reddy et al. proposed a method for optimizing the mechanical properties of concrete using graphene oxide [19]. To verify the performance of the research method, the mechanical properties of concrete optimized by two methods are compared, as shown in Table 2. In concrete with relatively heavy mechanical properties, the concrete mix ratio of the research method is: TP 10%, WCR 0.3, and APG 8:2.

**Table 2:** Comparison results of the mechanical properties of concrete.

Number	Traditional concrete		Oxidized graphene concrete (Reference [19])		Low carbon concrete (This study)	
	Resist compression (MPa)	Anti-folding (MPa)	Resist compression (MPa)	Anti-folding (MPa)	Resist compression (MPa)	Anti-folding (MPa)
1	38***	4.6***	62	8.2*	64	10.3
2	37***	4.5***	61	8.2*	65	10.2
3	38***	4.6***	63	8.1*	64	10.2
4	36***	4.7***	62	8.3*	63	10.3
5	38***	4.6***	61	8.3*	64	10.2

Note: *, and *** indicate that compared to the results of the research method, *p*<0.05 and *p*<0.01.

According to Table 2, the concrete optimized by the research method has higher CS and FS than traditional concrete. The CS of research concrete can reach over 60MPa, while traditional concrete can only maintain around 38MPa. The FS of research concrete can reach over 10MPa, while the FS of traditional concrete does not exceed 5MPa. Compared with the concrete designed in reference [19], the CS advantage of the research concrete is not significant, but the FS has been significantly improved.

### 4.2 Analysis of economic effects of RPC

RPC is a common green, low-carbon, and economical material, but buildings that use this material cannot become green buildings. The commonly used green building evaluation standards are used to evaluate RPC buildings, and the specific results are shown in Table 3.

**Table 3:** Evaluation standard for green buildings.

Evaluating indicator	Weight	Score	Final Score
Land conservation and outdoor environment	0.21	86	18.06
Energy conservation and utilization	0.24	75	18.00
Water conservation and water resource utilization	0.20	98	19.60
Material conservation and utilization of material resources	0.17	69	11.73
Indoor environmental quality	0.18	79	14.22
Extra points	1	5	5
Total score	/	/	86.61

**Fig 13:**
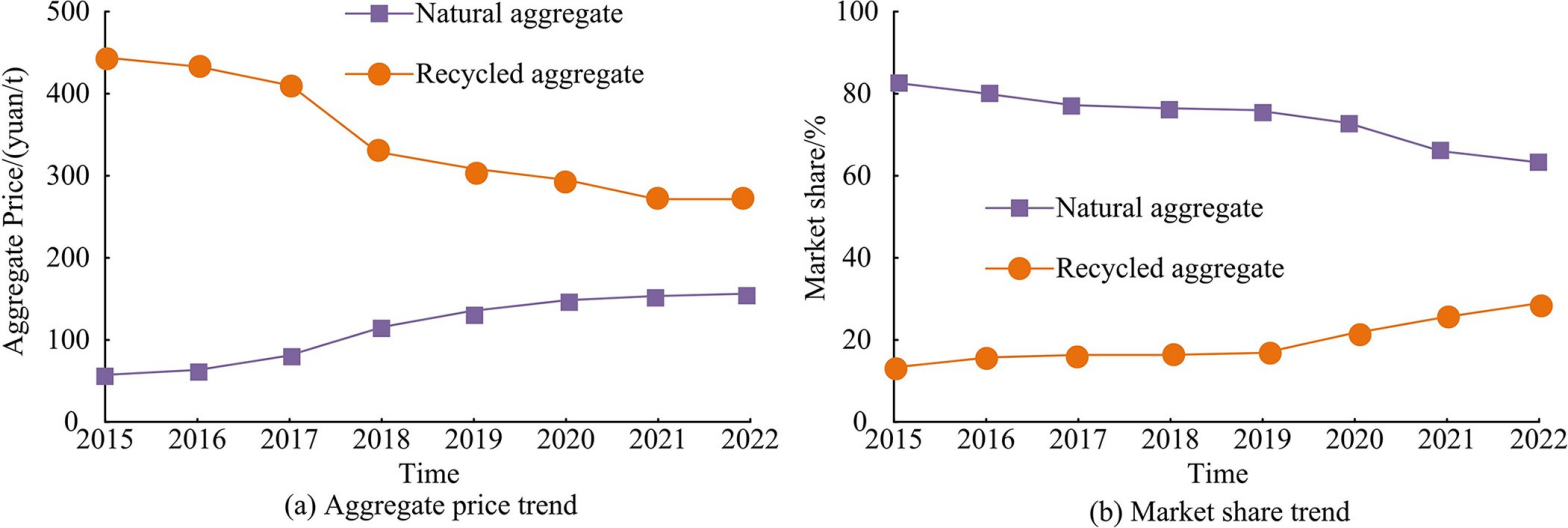
Comparison of aggregate price trends and application scope.

Table 3 shows that RPC materials can effectively meet the two indicators of land conservation and outdoor environment, water conservation and water resource utilization in buildings. If RPC materials are used as road paving materials and equipped with corresponding rainwater recycling systems, the recycling and utilization of rainwater resources in the region can be achieved. RPC has significant economic benefits for rainwater recovery systems. This study also analyzes the price trend of natural aggregate and recycled aggregate, as well as the market application share of natural aggregate and recycled aggregate, as shown in Fig 13.

Fig 13A shows the price trend of aggregates. Since 2015, the prices of natural aggregates have continued to rise, while the prices of recycled aggregates have shown a downward trend. From 2015 to 2022, the price of natural aggregate increased from 52.36 yuan/t to 148.59 yuan/t. The price of recycled aggregate decreased from 438.62 yuan/t to 236.48 yuan/t. From 2015 to 2022, the demand for the construction market increased significantly due to the acceleration of urbanization. Concurrently, there was a decrease in the reserves of natural aggregate. These two factors led to an increase in the price of natural aggregate and the continuous maturity of recycled aggregate-related technologies. The manufacturing of recycled aggregate was further promoted as a result. The price increase of natural aggregate was 183.8%, while the price increase of recycled aggregate was -46.1%. Fig 13B shows the market share of two types of aggregates. The market share of natural aggregates continues to decline, while the market share of recycled aggregates continues to rise. From 2015 to 2022, the market share of natural aggregates decreased from 82.3% to 64.9%, while the market share of recycled aggregates increased from 17.7% to 35.1%. The depletion of natural aggregate resources and the strengthening of environmental protection policies have led to an increase in the mining cost of natural aggregates. The advancement of recycled aggregate production technology and the promotion of recycling have reduced the production cost of recycled aggregates. In response to the concept of green development and to control production costs, the market share of recycled aggregates will continue to increase in the future. Adjusting the proportion of recycled aggregates in concrete structures can adapt to market development. The application of RPC research results in different geographical markets and architectural scenarios should consider regional economic level, environmental regulations, climate conditions, supply chain maturity, cultural aesthetics, construction technology, market demand, and sustainability goals. In economically developed areas, the environmental characteristics of RPC may be preferred, and its cost effectiveness in developing areas is notable. Under different climatic conditions, the RPC water permeability needs to adapt to the local needs. Regional supply chain conditions and construction capabilities will also affect the RPC implementation.

## 5. Conclusion

In response to the national low-carbon development concept, this study proposed to optimize the ratio of RPC to improve its mechanical properties and expand its application range. The optimization method for concrete mix proportion used in the study considered the volume method of APG and TP. This method determined the amount of material used through equations of actual porosity regarding APG and TP, CS regarding actual porosity, FS regarding actual porosity, and PC regarding APG and TP. The results showed that when TP was 10% and WCR was 0.28, the PC of concrete decreased by 0.87mm/s as PSD changed from 0:10 to 10:0. When TP was 14% and WCR was 0.28, the PC of concrete decreased to the lowest point when PSD was 8:2, and at this time, the PC of concrete was 2.14mm/s. When the concrete PSD was fixed, the PC of the concrete would increase with the increase of TP. When TP was 10% and WCR was 0.28, the PCoS was 2.53mm/s. When TP was 20%, the PCoS was 4.69mm/s, a total increase of 85.4%. As the APG level increased, the average PC showed a trend of first decreasing and then increasing. When the APG level was 4, the average PC decreased to the lowest. The price of recycled aggregate continued to decline, and the market share was also constantly increasing. The perspective coefficient of concrete was low when the APG was 8:2. The CS of the research concrete has reached more than 60MPa, much higher than the 38MPa of the traditional concrete. At the same time, the anti-folding strength of the research concrete could reach more than 10MPa, which was also significantly improved. This study focused on the effects of TP and APG on the permeability of concrete, with little attention paid to WCR. The study analyzed the interaction between WCR, TP, and APG variables and their impact on RPC performance, providing a deeper understanding of how these factors collectively affect concrete performance. This multivariate interaction analysis method could be applied to the study of other building materials to optimize material properties. The study also revealed the direct impact of aggregate PSD on the porosity of concrete. This indicates that when designing concrete mixtures, precise control of aggregate PSD can effectively regulate the pore structure of concrete, thereby affecting its permeability and strength. Future research can be conducted in this direction. RPC research design may face challenges in material quality control, cost-effectiveness, construction technology, market acceptance, policy regulations, environmental impact assessment, and other aspects in practical applications. To address these issues, strict aggregate screening procedures, reduced production costs, professional construction training, increased market awareness, cooperation with the government to promote policy formulation, and comprehensive environmental assessments are needed to ensure the successful implementation and promotion of RPC. In addition, further exploration of the interactions of water-cement ratio, porosity, and aggregate grading can provide more parameter suggestions for the implementation of expansion in practical situations.

## Supporting information

S1 File (DOCX)
