# Supplementary material for: Insight into the mechanical properties and the sustainable application of recycled permeable concrete: Green low-carbon concrete technology
Source: PLoS One. 2025 Feb 6;20(2):e0318684. doi: 10.1371/journal.pone.0318684 (PMC11801643; doi:10.1371/journal.pone.0318684)
Supplement: S1 File — (DOCX) [file pone.0318684.s001.docx]

Minimal Data Set Definition

Website: [https://journals.plos.org/plosone/s/data-availability#loc-minimal-data-set-definition](https://journals.plos.org/plosone/s/data-availability#loc-minimal-data-set-definition)

Authors must share the “minimal data set” for their submission. PLOS defines the minimal data set to consist of the data required to replicate all study findings reported in the article, as well as related metadata and methods. Additionally, PLOS requires that authors comply with field-specific standards for preparation, recording, and deposition of data when applicable.

For example, authors should submit the following data:

- The values behind the means, standard deviations and other measures reported;

- The values used to build graphs;

- The points extracted from images for analysis.

The raw data are as follows:

Figure 7：

| APG | TP | WCR | PC (mm/s) |
| --- | --- | --- | --- |
| 0:10 | 10% | 0.28 | 2.53 |
| 10:0 | 10% | 0.28 | 1.66 |
| 0:10 | 10% | 0.30 | 2.75 |
| 10:0 | 10% | 0.30 | 1.82 |
| 0:10 | 10% | 0.32 | 3.88 |
| 10:0 | 10% | 0.32 | 2.61 |
| 8:2 | 12% | 0.28 | 1.55 |
| 10:0 | 12% | 0.28 | 1.73 |
| 8:2 | 12% | 0.30 | 1.89 |
| 10:0 | 12% | 0.30 | 1.96 |
| 8:2 | 12% | 0.32 | 2.64 |
| 10:0 | 12% | 0.32 | 3.21 |

Figure 8:

| APG | TP | WCR | PC (mm/s) |
| --- | --- | --- | --- |
| 0:10 | 14% | 0.28 | 2.14 |
| 10:0 | 14% | 0.28 | 2.37 |
| 8:2 | 14% | 0.28 | 1.91 |
| 10:0 | 14% | 0.30 | 2.06 |
| 8:2 | 14% | 0.30 | 1.80 |
| 10:0 | 14% | 0.32 | 2.11 |
| 8:2 | 14% | 0.32 | 1.61 |
| 0:10 | 16% | 0.28 | 2.59 |
| 10:0 | 16% | 0.28 | 2.81 |
| 8:2 | 16% | 0.28 | 2.34 |
| 10:0 | 16% | 0.30 | 2.46 |
| 8:2 | 16% | 0.30 | 2.15 |

Figure 9:

| PSD | TP | WCR | PC (mm/s) |
| --- | --- | --- | --- |
| 0:10 | 10% | 0.28 | 2.53 |
| 0:10 | 20% | 0.28 | 4.69 |
| 2:8 | 10% | 0.28 | 2.18 |
| 2:8 | 20% | 0.28 | 4.18 |
| 0:10 | 10% | 0.30 | 2.75 |
| 0:10 | 20% | 0.30 | 5.33 |
| 2:8 | 10% | 0.30 | 2.66 |
| 2:8 | 20% | 0.30 | 4.46 |

Figure 10:

| PSD | TP | WCR | PC (mm/s) |
| --- | --- | --- | --- |
| 4:6 | 10% | 0.28 | 1.75 |
| 4:6 | 20% | 0.28 | 3.51 |
| 6:4 | 10% | 0.28 | 1.42 |
| 6:4 | 20% | 0.28 | 3.37 |
| 4:6 | 10% | 0.30 | 1.79 |
| 4:6 | 20% | 0.30 | 4.27 |
| 6:4 | 10% | 0.30 | 1.24 |
| 6:4 | 20% | 0.30 | 3.79 |

Figure 11:

| PSD | TP | WCR | PC (mm/s) |
| --- | --- | --- | --- |
| 8:2 | 10% | 0.28 | 1.35 |
| 8:2 | 20% | 0.28 | 1.73 |
| 10:0 | 10% | 0.28 | 2.63 |
| 10:0 | 20% | 0.28 | 3.28 |
| 8:2 | 10% | 0.30 | 1.63 |
| 8:2 | 20% | 0.30 | 2.46 |
| 10:0 | 10% | 0.30 | 2.89 |
| 10:0 | 20% | 0.30 | 3.50 |
